# Cerebrospinal fluid neurofilament light chain in acute optic neuritis and its predictive ability of multiple sclerosis

**DOI:** 10.1007/s00415-024-12587-8

**Published:** 2024-07-25

**Authors:** Moschoula Passali, Ian Galea, Maria Højberg Knudsen, Laurie Chi Lau, Stig Præstekjær Cramer, Jette Lautrup Frederiksen

**Affiliations:** 1 Optic Neuritis Clinic, Danish Multiple Sclerosis Center, Department of Neurology, Copenhagen University Hospital Rigshospitalet-Glostrup, Glostrup, Denmark; 2 Department of Clinical Medicine, Faculty of Health and Medical Science, University of Copenhagen, Copenhagen, Denmark; 3 Clinical Neurosciences, Clinical and Experimental Sciences, Faculty of Medicine, University of Southampton, Southampton, UK; 4 Functional Imaging Unit, Department of Clinical Physiology, Nuclear Medicine and PET, Copenhagen University Hospital Rigshospitalet-Glostrup, Glostrup, Denmark

**Keywords:** Multiple sclerosis, Optic neuritis, Clinically isolated syndrome, Neurofilament light chain, Biomarkers, Early diagnosis

## Abstract

**Background:**

Studies on the capability of cerebrospinal fluid neurofilament light chain (cNfL) to predict multiple sclerosis (MS) conversion in clinically isolated syndromes have yielded varying results.

**Objectives:**

To expand our understanding of cNfL in optic neuritis (ON) and investigate whether incorporating cNfL into the 2017 McDonald criteria could accelerate the diagnosis of MS in patients with ON.

**Methods:**

cNfL was measured in diagnostic samples from 74 patients with verified ON. MS was diagnosed using the 2017 McDonald criteria with a minimum observation time of two years from ON onset.

**Results:**

20.5% of 44 MS-converters did *not* fulfil the 2017 McDonald criteria at ON onset. A doubling of cNfL was associated with 207% (74%–514%) higher odds of MS (p = 0.00042, adjusted for age). Fulfilment of ≥ 1 MRI criterion for dissemination in space (DIS) and presence of brain contrast-enhancing lesions were associated with higher cNfL. Furthermore, cNfL correlated with inter-eye differences in retinal nerve fiber layer (RNFL) thickness (Spearman’s ρ = 0.46, p = 8 × 10^–5^). Incorporating cNfL ≥ 906 pg/mL as a substitute for either dissemination in time or one MRI criterion for DIS increased the sensitivity (90.9% vs. 79.6%) and accuracy (91.9% vs. 87.8%), but also reduced the specificity (93.3% vs. 100%) of the 2017 McDonald criteria.

**Conclusion:**

cNfL was related to MS diagnostic parameters and the degree of RNFL swelling. Clinical use of cNfL may aid in identification of ON patients with increased risk of MS until larger studies have elaborated on the potential loss of specificity if used diagnostically.

## Introduction

In multiple sclerosis (MS), early treatment initiation with high efficacy treatments is associated with lower rates of disability highlighting the importance of early diagnosis of MS among patients with clinically isolated syndromes (CIS) [1, 2]. MS is currently diagnosed using the 2017 McDonald criteria [3]. They are based on demonstration of dissemination in space (DIS) and dissemination in time (DIT) in patients with typical clinical episode(s), and no better explanation for the clinical presentation [3]. It has been estimated that 19% of patients with MS present with optic neuritis (ON) as their first CIS [4]. Introduction of oligoclonal bands (OCB) as a substitute for DIT allowed for earlier diagnosis of MS [5], however, there are still patients that do not fulfill the 2017 McDonald criteria at their first ON, who later go on to develop MS.

Although great progress has been made towards clinical application of blood NfL as a biomarker of disease activity and progression in MS, cerebrospinal fluid NfL (cNfL) has received much less attention in MS research during the last years [6]. Concentrations of cNfL have consistently been found to be increased already early in the disease course of MS compared to controls [7–14]. Conversely, studies examining the predictive capability of cNfL for MS-conversion among patients with CIS have yielded variable results both with regards to statistical significance and effect sizes [8–18]. Use of different diagnostic criteria as well as examination of different forms of CIS are potential explanations for this variation.

No studies have evaluated the potential of cNfL to improve the diagnostic ability of the 2017 McDonald criteria, however, a multicenter study on blood NfL found promising results [19]. We hypothesize that the ability of cNfL to predict MS-conversion, may be higher in ON compared to other forms of CIS. This is because the brain and/or spinal cord are expected to be the main source of cNfL in non-ON CIS patients independent of MS conversion, whereas the optic nerve is expected to be the primary source of cNfL in patients with idiopathic ON. The aim of this study is to evaluate the potential of cNfL, which is more sensitive than blood NfL, to predict MS-conversion according to the 2017 McDonald criteria in a homogeneous cohort of patients with verified ON.

## Materials and methods

### Study participants

This analysis is performed on baseline data from a clinical trial studying the effects of a gluten-free diet in patients with ON (ClinicalTrials.gov identifier: NCT03451955). From January 2018 to November 2021, 216 patients were consecutively referred to the Clinic of Optic Neuritis at Copenhagen University Hospital, Rigshospitalet-Glostrup. All referred patients with suspected ON, that were aged 18–59 years old and were deemed mentally and physically able to participate in the trial were invited to do so. Exclusion criteria were pregnancy or lactation, confounding comorbidities, surgery within the last 6 weeks, presence of MRI incompatible implants, severe claustrophobia, previous reactions to MRI contrast agent, severe allergies, elevated serum creatinine or bronchial asthma. Out of 90 enrolled patients with a verified ON who had completed the baseline examinations of the clinical trial, eight were excluded due to a differential diagnosis (seven had myelin oligodendrocyte glycoprotein (MOG) antibody disease and one had sarcoidosis), whereas CSF was not available for measurement of cNfL from eight patients. The remaining 74 patients were included in this analysis. All patients were untreated at the time of lumbar puncture. A professor of neurology with specialty in ON and MS verified the diagnosis of ON and diagnosed patients with MS using the 2017 McDonald criteria. The test battery used to verify ON and exclude differential diagnoses included tests of low and high contrast visual acuity, tests of color vision, optical coherence tomography (OCT) and visual evoked potentials in the acute phase in addition to extensive blood tests (aquaporin-4 antibodies, MOG antibodies, anti-nuclear antibodies, angiotensin converting enzyme etc.), chest X-ray as well as ophthalmological and neurological history and examination. All patients were offered follow-up MRI and lumbar puncture at six months post recruitment as part of their participation in the clinical trial. In addition, ON patients were offered yearly MRIs for at least two years following ON onset. Minimal observation time with regards to MS diagnosis was 24 months (range: 24–69 months). The professor of neurology diagnosing the patients was blinded to the results of the cNfL analyses.

### CSF analyses

CSF was obtained on ice, centrifuged as soon as possible (400×*g* for 10 min at 4 °C), aliquoted into 500 μL microcentrifuge tubes and stored at −80 °C. Measurements of cNfL were performed in duplicates using enzyme-linked immunosorbent assay (Uman Diagnostics) according to the manufacturer instructions. Detection of mismatched oligoclonal bands (OCB), and measurements of IgG-index and CSF leukocyte concentrations were performed as part of routine analyses of CSF from diagnostic lumbar punctures in our clinic. OCB were considered present when at least two IgG bands were detected in CSF but not in serum. OCB were visualized by isoelectric focusing with immunoblotting. Questionable OCB were rerun. All personnel performing fluid biomarker analyses were blinded towards the patients’ potential diagnosis of MS, medical history and results from MRI analyses. Data on IgG-index and CSF leukocyte concentration were not included from 1 patient due to erythrocyte contamination. In case of values below the limit of quantification of CSF leukocytes (3 million/L, n = 22) and CSF IgG (10 mg/L, n = 3), CSF leukocytes were set to 2 million/L, whereas a CSF IgG value of 8 mg/L was used in the calculation of the IgG-index.

### Magnetic resonance imaging

All patients underwent contrast-enhanced magnetic resonance imaging (MRI) of the brain and cervical cord that was of diagnostic quality. In all but three patients, MRI was performed using a Philips Achieva 3 T scanner. Contrast enhancement was evaluated in the brain but not in the cervical cord. One patient declined to receive contrast agent but achieved MS diagnostic criteria at presentation. The number of lesions in the brain (categorized as 0–1, 2–8 & ≥ 9), the number of criteria for dissemination in space and the presence of contrast enhancement were assessed by a specialist of MRI in MS with more than 30 years of experience. The MRI specialist was blinded to cNfL and other fluid biomarker results.

### Optical coherence tomography

OCT was performed using Cirrus 4000 HD-OCT (Carl-Zeiss). Inter-eye retinal nerve fiber layer (RNFL) thickness was calculated as RNFL of the affected eye minus RNFL of the unaffected eye. Data on inter-eye RNFL thickness were excluded from four patients with past ON and one patient with bilateral ON.

### Statistics

Descriptive statistics are presented as number with respective percentage for categorical variables and median with interquartile range for non-normally distributed numerical variables. The Shapiro–Wilk normality test was used to evaluate the normality of distributions. There were no missing data with regards to OCB, MRI criteria for DIS, diagnosis or age. Mann–Whitney U test was used to test for differences between numerical, non-normally distributed variables. cNfL values were transformed using the natural logarithm in linear regression models and the logarithm with base 2 in logistic regression models. All linear regression and logistic regression models including cNfL were adjusted for age. Receiver operating characteristic (ROC) curves were used to evaluate the performance of logistic regression models with MS-conversion at the end of the observation time as dependent variable. Areas under the curve (AUC) with their respective 95% confidence intervals are presented. The Akaike information criterion (AIC) is provided for all logistic regression models. DeLong’s test for two correlated ROC curves was used to test for a difference between ROC curves. Selection of cut-off value was based on Youden’s index. Analyses were performed in R versions 4.3.1 and 4.3.2 [20] using packages readxl [21], dplyr [22], table 1[23], ggplot2 [24], ggpubr [25] and pROC [26].

**Table 1:** Baseline characteristics of optic neuritis cohort

	All ON (n = 74)	Non-converters (n = 30)	Early MS-converters (n = 35)	Late MS-converters (n = 9)
Gender, female	48 (64.9%)	19 (63.3%)	22 (62.9%)	7 (77.8%)
Age (years)	32 [27, 39]	33.5 [27, 36]	31 [26.5, 43.5]	32 [29, 38]
History of neurologic symptoms	6 (8.1%)	3 (10.0%)	3 (8.6%)	0 (0%)
Time from ON onset to LBP (days)	29 [22, 41]	30 [23, 39]	28 [20, 42]	38 [22, 67]
Time from MRI to LBP (days)	7 [3, 13]	8 [4, 12]	6 [3, 12]	8 [1, 18]
Brain white matter lesions (n)				
0–1	31 (41.9%)	23 (76.7%)	2 (5.7%)	6 (66.7%)
2–8	22 (29.7%)	6 (20.0%)	14 (40.0%)	2 (22.2%)
≥ 9	21 (28.4%)	1 (3.3%)	19 (54.3%)	1 (11.1%)
Presence of brain CE-lesions	13 (17.8%)^n=73^	0 (0%)	11 (32.4%)^n=34^	2 (22.2%)
Presence of oligoclonal bands	53 (71.6%)	11 (36.7%)	35 (100%)	7 (77.8%)
Leukocytes in CSF (10^6^/L)^a^	5 [2, 8]	3 [2, 6]	6 [4, 9.8]^n=34^	5 [2, 16]
IgG-index^a^	0.58 [0.47, 0.79]	0.48 [0.43, 0.54]	0.69 [0.57, 0.92]^n=34^	0.66 [0.54, 0.96]

^a^CSF leukocyte values are considered abnormal when ≥ 5·10^6^/L, whereas IgG-index values are considered increased when > 0.67. Categorical variables are presented as number (percentage). Non-normally distributed variables are presented as median (1^st^ quartile, 3^rd^ quartile). The term non-converters refers to patients with idiopathic ON who did not fulfill the 2017 McDonald criteria for MS during the observation time of the study (minimum 2 years). Early MS-converters are ON patients who received the diagnosis of MS at ON onset. Late MS-converters are ON patients who did not receive the diagnosis of MS at ON onset but fulfilled the 2017 McDonald criteria within the observation time of the study

*CE* contrast-enhancing, *CSF* cerebrospinal fluid, *IgG* Immunoglobulin G, *LBP* lumbar puncture, *MRI* magnetic resonance imaging, *MS* multiple sclerosis, *ON* optic neuritis

## Results

### Patient characteristics

Demographic characteristics as well as results from MRI and CSF analyses of the 74 ON patients are presented in Table 1. Three ON patients had historical evidence of previous ON, but did not receive the diagnosis of MS (absence of OCB and zero MRI criteria for DIS). In addition, three patients had historical evidence of previous ON, sensory and/or motor relapses and received the diagnosis of MS at ON onset (presence of OCB and DIS on MRI). All patients with brain contrast-enhancing (CE) lesions had OCB. By the end of the observation time, the MS conversion rate was 59.5%. Out of the 44 MS-converters, 20.5% did not fulfill the 2017 McDonald criteria at ON onset but received the diagnosis MS within the observation time of the study (late MS-converters). At baseline, DIS was demonstrated in all early MS-converters, 11.1% of late MS converters and 0% of non-converters. Similarly, DIT or OCB were demonstrated in all early MS-converters, 77.8% of late MS-converters and 46.7% of non-converters. Treatment initiation was not affected by study participation. All patients were untreated at the time of lumbar puncture (no use of steroids or disease-modifying treatments). Only two patients were initiated on disease-modifying treatment (dimethyl fumarate, Tecfidera) prior to fulfilling the 2017 McDonald criteria. Both received the diagnosis of MS during the observation time of the study.

### Relationship between cNfL, MS diagnostic parameters and ON severity

**Fig. 1:**
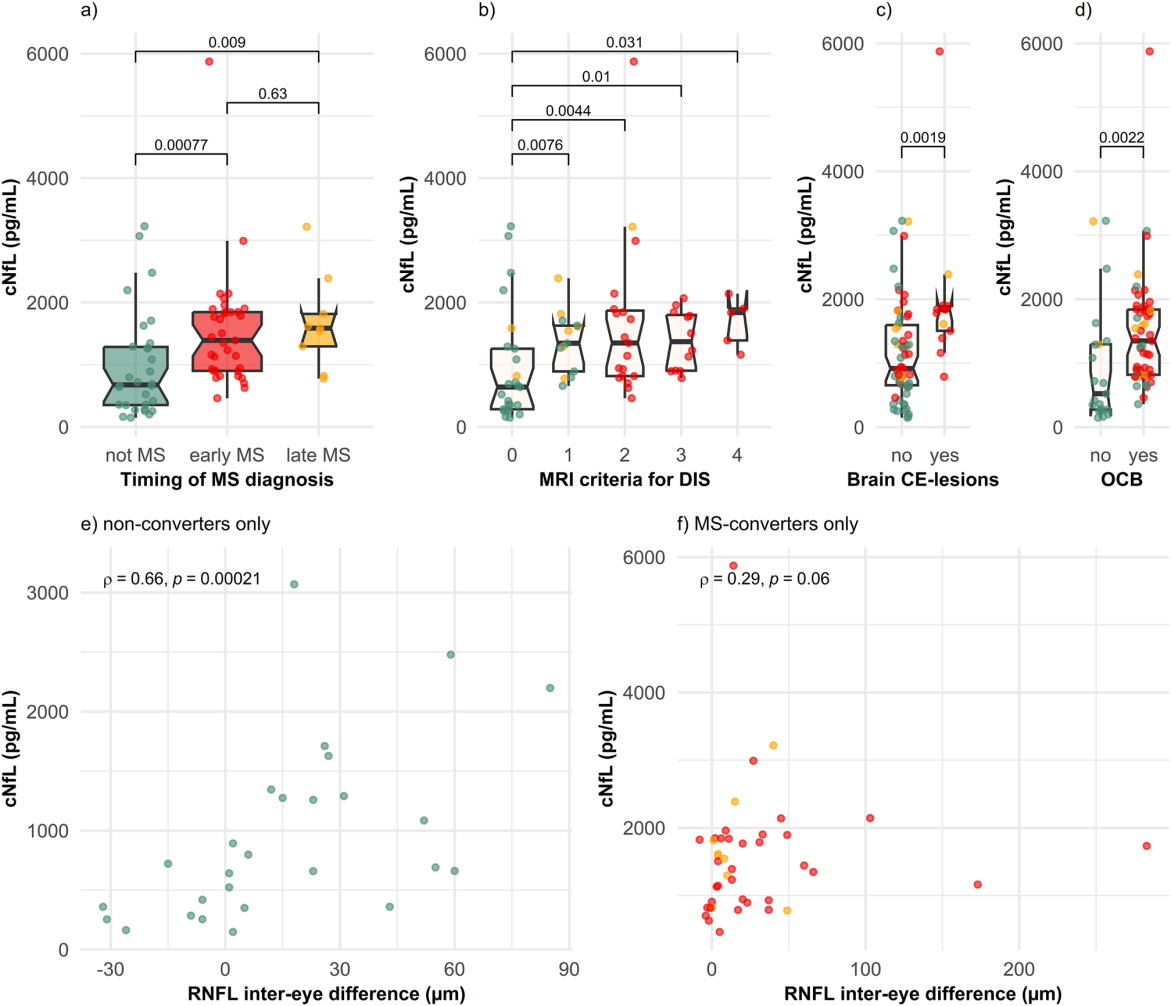
cNfL concentrations in patients with ON according to the timing of MS diagnosis (**a**), the number of fulfilled MRI criteria for dissemination in space (**b**), the presence of brain CE-lesions (**c**), the presence of OCB (**d**) and in correlation with inter-eye difference in RNFL (**e**) at ON onset. Statistical analyses were performed using Mann–Whitney U test and Spearman’s correlation. Datapoints are colored according to timing of MS diagnosis: green = “not MS”, red = “early MS”, orange = “late MS”. The term “not MS” refers to patients with idiopathic ON who did not fulfill the 2017 McDonald criteria for MS during the observation time of the study (minimum 2 years). “Early MS” refers to ON patients who received the diagnosis of MS at ON onset. “Late MS” refers to ON patients who did not receive the diagnosis of MS at ON onset but fulfilled the 2017 McDonald criteria within the observation time of the study. *CE* contrast-enhancing, *cNfL* cerebrospinal fluid neurofilament light chain, *DIS* dissemination in space, *MRI* magnetic resonance imaging, *MS* multiple sclerosis, *OCB* oligoclonal bands, *ON* optic neuritis, *RNFL* retinal nerve fiber layer

Non-converters had significantly lower cNfL compared to early MS-converters fulfilling the 2017 McDonald criteria at ON onset (676 [352, 1290] vs. 1390 [903, 1850], p = 0.00077, Mann–Whitney U test) and late MS-converters (676 [352, 1290] vs. 1590 [1300, 1820], p = 0.009, Mann–Whitney U test) (Table 1 and Fig. 1a). In a linear regression model with log-transformed cNfL as dependent variable and age and diagnosis as independent variables, MS converters had 97% (45%–169%) higher cNfL (p = 3.6 × 10^–5^) than non-converters and cNfL increased by 2% (0.3%-3.8%) for each additional year of age (p = 0.020). cNfL correlated with age in non-converters (Spearman’s ρ = 0.46, p = 0.0097) and late MS-converters (Spearman’s ρ = 0.74, p = 0.024), but not in early MS-converters (Spearman’s ρ = 0.0081, p = 0.96).

To investigate which MS-related diagnostic parameters were associated with cNfL, we plotted the unadjusted relationships with MRI criteria for DIS (Fig. 1b), brain CE-lesions (Fig. 1c) and OCB (Fig. 1d). Interestingly, fulfilling 0 MRI criteria for DIS was associated with 52% lower (25%-69%) cNfL compared to 1 MRI criterion for DIS (p = 0.0016, linear regression adjusted for age). However, having dissemination in more than 1 CNS site was not associated with further increases in cNfL. Subgroup analysis revealed that non-converters with 1 MRI criterion for DIS also had higher cNfL compared to non-converters with 0 MRI criteria for DIS (1274 [845–1487] vs. 523 [280–1172], p = 0.048, Mann–Whitney U test). Brain CE-lesions were only present in MS-converters (Fig. 1c) and MS-converters with brain CE-lesions had increased cNfL when compared to MS-converters without brain CE-lesions (1839 [1507, 1894] vs. 1188 [839, 1759], p = 0.018, Mann–Whitney U test). Similarly, only two MS converters did not have OCB at ON onset (Fig. 1d). When solely examining non-converters, there was only a trend towards higher cNfL in non-converters with OCB compared to non-converters without OCB (892 [660, 1492] vs. 418 [265, 1188], p = 0.064, Mann–Whitney U test). Co-occurrence of OCB and 1 MRI criterion for DIS was observed in 16.7% of non-converters.

We hypothesized that part of the remaining variation in cNfL could potentially be explained by differences in the degree of axonal damage occurring at the optic nerve. In the acute phase of ON, increased inter-eye differences in RNFL can be observed, illustrating the presence of edema in the RNFL of the affected eye [27]. Inter-eye differences in RNFL did indeed correlate with cNfL (Spearman’s ρ = 0.46, p = 8 × 10^–5^). When examined according to the timing of MS diagnosis, we observed that the correlation between inter-eye differences in RNFL and cNfL was strongest among non-converters (Spearman’s ρ = 0.66, p = 0.00021, Fig. 1e) compared to early MS-converters (Spearman’s ρ = 0.35, p = 0.049, Fig. 1e) and late MS-converters (Spearman’s ρ = 0.075, p = 0.85, Fig. 1e). In a multivariate linear regression model based on non-converters only, with log-transformed cNfL as dependent variable and inter-eye differences in RNFL, age, fulfilment of 1 MRI criterion for DIS (yes/no) and presence of OCB as independent variables, cNfL increased by 1.3% (0.3%–2.2%) for each additional µm in inter-eye RNFL difference (p = 0.013).

### cNfL and risk of MS

**Fig. 2:**
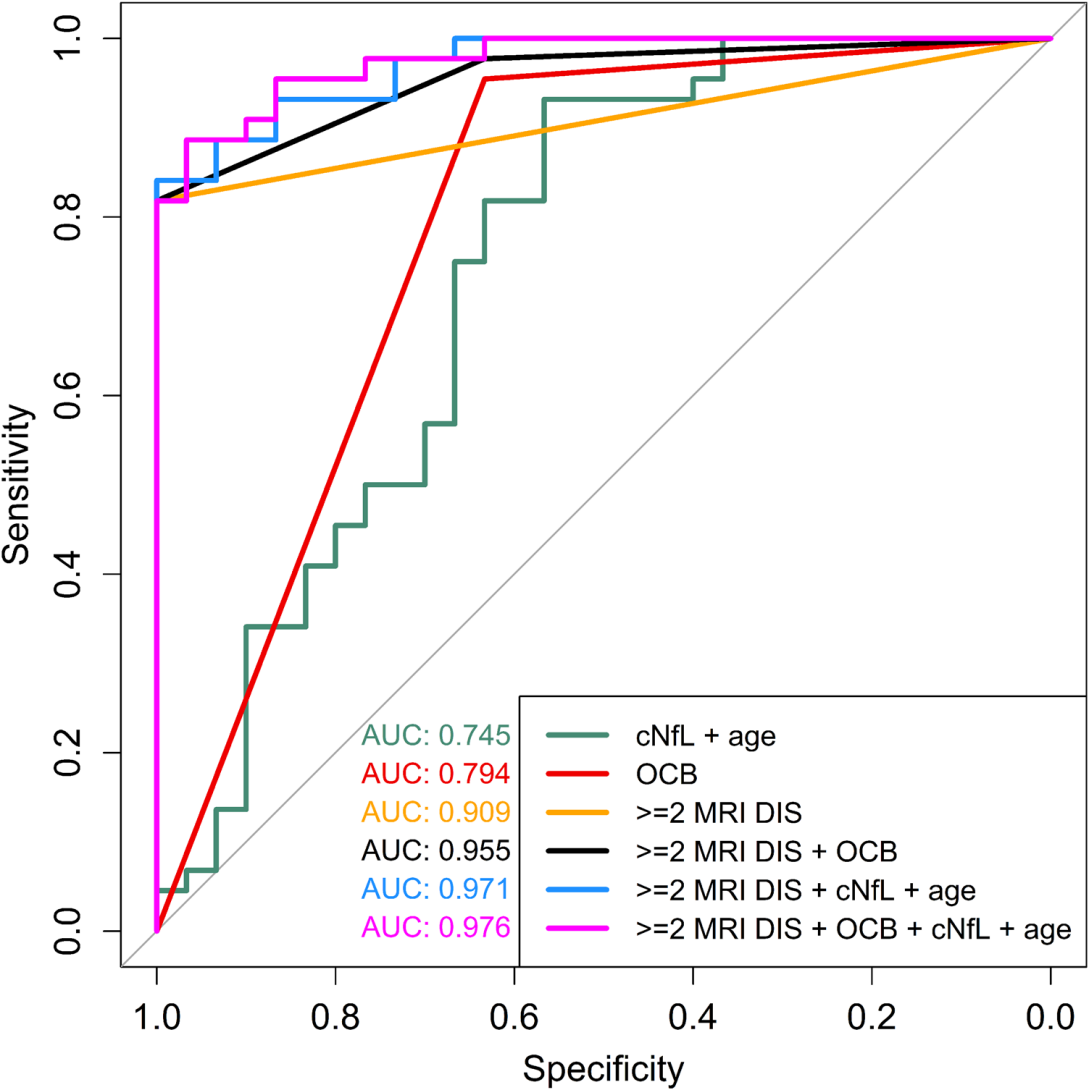
Receiver operating characteristic curves of logistic regression models with MS diagnosis (2017 McDonald criteria) by the end of the observation time as dependent variable. Independent variables comprise log2-transformed cNfL, age, OCB and MRI criteria for DIS (categoric variable: ≥ 2 or < 2) at ON onset. *AUC* area under the curve, *cNfL* cerebrospinal fluid neurofilament light chain, *DIS* dissemination in space, *MRI* magnetic resonance imaging, *MS* multiple sclerosis, *OCB* oligoclonal bands

In a logistic regression model with MS diagnosis at the end of the observation time as dependent variable and log2-transformed cNfL and age as independent variables, a doubling of cNfL was associated with 207% (74%–514%) higher odds of MS (OR = 3.07, 95% CI (1.74–6.14), p = 0.00042, AIC = 88.30) (Fig. 2). When further adding the presence of ≥ 2 MRI criteria for DIS as an independent variable (yes/no) to the above model, the respective ROC analysis reached an AUC of 0.971 (95% CI (0.942–1), AIC = 37.24). As a comparison, the logistic regression model representing the 2017 McDonald criteria with the presence of ≥ 2 MRI criteria for DIS (yes/no) and OCB as independent variables had an AUC of 0.955 (95% CI (0.917–0.994), AIC = 38.00). To evaluate whether incorporation of cNfL has the potential to improve the 2017 McDonald criteria, we created a multivariate logistic regression model with MS diagnosis at the end of the observation time as dependent variable and the presence of ≥ 2 MRI criteria for DIS (yes/no), OCB, log2-transformed cNfL and age as independent variables. This last model had an AUC of 0.976 (95% CI (0.950–1), AIC = 36.95), however its comparison to the model representing the 2017 McDonald criteria did not reach statistical significance (p = 0.0755, DeLong’s test for two correlated ROC curves). An overview of ROC analyses is presented in Fig. 2.

Next, we examined how different scenarios of incorporating cNfL into the 2017 McDonald criteria would affect their sensitivity, specificity and accuracy (Table 2). As our dataset was not large enough to identify age-specific cut-off values, we defined a universal optimal cut-off for cNfL at 906 pg/mL corresponding to the value with the maximum Youden’s index (0.83) in a multivariate model with cNfL, OCB and presence of ≥ 2 MRI criteria for DIS (yes/no) as independent variables. cNfL values were equal to or above the selected threshold in 59.5% of all ON patients (77.8% of late MS converters, 74.3% of early MS-converters and 36.7% of non-converters). Inclusion of cNfL ≥ 906 pg/mL as a substitute for DIT in patients with ≥ 2 MRI criteria for DIS resulted in minor increases in sensitivity and accuracy without affecting the specificity of the 2017 McDonald criteria (Table 2). However, when cNfL ≥ 906 pg/mL was incorporated as a substitute for 1 MRI criterion of DIS in patients with OCB, specificity was reduced to 93.3%. In a combinatorial approach, use of cNfL ≥ 906 pg/mL as a substitute for either DIT or 1 MRI criterion of DIS increased the accuracy of the 2017 McDonald criteria from 87.8% to 91.9% (Table 2).

**Table 2:** Sensitivity, specificity and accuracy estimates of different scenarios for incorporating cNfL into the 2017 McDonald criteria

	Sensitivity	Specificity	Accuracy
*Univariate*			
≥ 1 MRI DIS	95.5%	76.7%	87.8%
≥ 2 MRI DIS	81.8%	100%	89.2%
OCB	95.5%	63.3%	82.4%
cNfL ≥ 906 pg/mL	79.5%	63.3%	70.3%
*Multivariate*			
2017 McDonald criteria^a^: ≥ 2 MRI DIS **AND** OCB	79.6%	100%	87.8%
cNfL as substitute for DIT: ≥ 2 MRI DIS **AND** (OCB **OR** cNfL ≥ 906 pg/mL)	81.8%	100%	89.2%
cNfL as substitute for 1 MRI DIS: (≥ 2 MRI DIS **AND** OCB) **OR** (≥ 1 MRI DIS **AND** OCB **AND** cNfL ≥ 906 pg/mL)	88.6%	93.3%	90.5%
cNfL as substitute for 1 MRI DIS or DIT: ≥ 2 MRI DIS **AND** (OCB **OR** cNfL ≥ 906 pg/mL) **OR** (≥ 1 MRI DIS **AND** OCB **AND** cNfL ≥ 906 pg/mL)	90.9%	93.3%	91.9%

^a^All patients with history of neurologic symptoms had either ≥ 2 MRI DIS and OCB or 0 MRI DIS and no OCB. Furthermore, all patients with brain contrast-enhancing lesions had OCB. Therefore, the 2017 McDonald criteria can accurately be represented by ≥ 2 MRI DIS and OCB in this cohort of patients with verified ON and no better explanation for the clinical presentation

*cNfL* cerebrospinal fluid neurofilament light chain, *DIS* dissemination in space, *DIT* dissemination in time, *MRI* magnetic resonance imaging, *OCB* oligoclonal bands

## Discussion

In this study including ON as the only form of CIS, we found that patients who fulfilled the 2017 McDonald criteria at ON onset had higher cNfL compared to patients who did not fulfil the 2017 McDonald criteria within the observation time of the study being minimum 2 years. More importantly, late MS-converters who did not fulfill the 2017 McDonald criteria at ON onset, also had higher cNfL compared to non-converters and their levels were equivalent to those of early MS-converters. The observed difference in cNfL levels between MS-converters and non-converters has previously been demonstrated in some [8, 11, 15–18] but not all [9, 10, 12, 13] studies of patients with CIS. Use of different MS diagnostic criteria, different follow-up times with regards to MS diagnosis, different methodologies to quantify cNfL as well as different sample sizes are potential explanations for this variation.

Additionally, we hypothesize that levels of cNfL may be higher in non-converters with CIS affecting the brain and/or the spinal cord compared to non-converters with ON. Although we did not have data from patients with other forms of CIS to explore this hypothesis, we did see that ON non-converters fulfilling 1 MRI criterion for DIS had higher cNfL compared to ON non-converters with 0 MRI criteria for DIS. Furthermore, we observed a moderate to strong correlation between inter-eye differences in RNFL thickness and cNfL in ON non-converters which was less pronounced in MS-converters. This could suggest that axonal damage in the optic nerve is probably the primary source of cNfL in most ON non-converters with other co-occurring mechanisms being considerable contributors in MS-converters. The relationship between cNfL and inter-eye RNFL thickness in the acute phase of ON has not been documented before, however, in a previous study from our clinic, baseline cNfL predicted visual outcome and inter-eye difference in RNFL thickness at six months follow-up after ON [28].

Our observation that 77.8% of late MS converters had OCB but did not demonstrate the necessary minimum of 2 MRI criteria for DIS suggests that—at least in ON—there is a greater need for biomarkers predictive of future DIS than DIT. Surprisingly, although fulfilment of ≥ 1 MRI criterion was associated with higher cNfL, we observed no dose–response relationship between cNfL and the number of MRI criteria for DIS. Biologically, this could suggest that older lesions which developed subclinically prior to ON onset are no longer contributing to the pool of cNfL. When examining the relationship between cNfL and OCB, non-converters with OCB did not have significantly higher cNfL than non-converters without OCB. Although, lack of statistical significance could be due to smaller sample sizes in subgroups, any potential contributions of intrathecal antibody synthesis to the pool of cNfL would still be much smaller than that of CNS lesions and RNFL swelling.

cNfL was a weaker individual predictor of MS than OCB and MRI criteria for DIS. However, when combined with the presence of ≥ 2 MRI criteria for DIS, cNfL was at least as strong a predictor of MS as OCB. Addition of cNfL and age to the logistic regression model representing the 2017 McDonald criteria, non-significantly increased the AUC of the respective ROC curve by 0.021. Although the increase is rather small in a statistical setting, it could still be of clinical relevance when considering that incorporation of OCB into the McDonald criteria had a considerable clinical impact, while addition of OCB to ≥ 2 MRI criteria for DIS only increased the AUC by 0.046 in our dataset.

Fulfilment of ≥ 2 MRI criteria for DIS had a specificity of 100% for MS diagnosis and using cNfL ≥ 906 pg/mL as a substitute for DIT slightly increased sensitivity without affecting the specificity of the 2017 McDonald criteria. On the contrary, using cNfL ≥ 906 pg/mL as a substitute for 1 MRI criterion for DIS, increased sensitivity and accuracy but also reduced specificity. It is well-established that increased cNfL is not specific for MS [29, 30]. Notably, when examined as individual predictors of MS in our dataset, the specificity of cNfL ≥ 906 pg/mL was identical to that of OCB which were present in 36.7% of non-converters. This is in line with the 2017 McDonald criteria having higher sensitivity and lower specificity than the 2010 McDonald criteria [5].

This study was performed on baseline data from consecutively recruited patients with verified ON participating in a clinical trial. Although, the inclusion and exclusion criteria of the trial were largely aligned with the target population of this study, we cannot rule out the possibility of a slight selection bias related to patients’ willingness to participate in a clinical trial which is more demanding than participation in observational biomarker studies. Low sample size is a weakness of this study and hindered us from determining age-specific cut-off values for cNfL. The positive correlation between age and cNfL in non-converters but not in early MS-converters suggests that the diagnostic value of cNfL may be highest in younger populations of acute ON. This could be evaluated in larger multicenter studies.

In conclusion, our data support that age, presence of lesions in at least one CNS site, presence of brain CE-lesions and increased inter-eye RNFL thickness are important contributors to the pool of cNfL in acute ON. Inclusion of cNfL as a substitute for DIT and/or 1 MRI criterion for DIS has the potential to increase the sensitivity and accuracy of the 2017 McDonald criteria in ON, however, this may also be associated with a loss of specificity. Until more precise specificity estimates have been provided by larger cohorts, we suggest that cNfL is used clinically to identify ON patients with increased risk of MS where more frequent monitoring would be sensible. Development of age-specific cut-offs could further increase the clinical value of cNfL measured in diagnostic lumbar punctures of patients with ON.

## Data Availability

The anonymized data used in this study can be provided by the corresponding author upon reasonable request.
